# Gangliosides in Cutaneous Melanoma: Molecular Mechanisms, Translational Evidence Gaps, and Preclinical Natural-Compound Leads

**DOI:** 10.3390/ijms27209021

**Published:** 2026-10-12

**Authors:** Deog-Bon Koo, Kyongtae Kim, Juhwan Kim, Hyo Gyeong Yun, Eun Bin Song, Min Young Kim, Jae-Sung Ryu, Ji-Su Kim, Soyeon Kim, Hyun-A Lee, Young-Kug Choo

**Affiliations:** 1 Department of Biotechnology, College of Engineering, Daegu University, 201 Deagudae-ro, Jillyang, Gyeongsan 38453, Gyeongbuk, Republic of Korea; 2 Department of Biological Science, College of Health Sciences, Wonkwang University, 460 Iksan-daero, Iksan-si 54538, Jeonbuk, Republic of Korea; 3 Division of Biodrug Evaluation, New Drug Development Center, Osong Medical Innovation Foundation (K-12 Bio Health), Cheongju 28160, Chungbuk, Republic of Korea; 4 Futuristic Animal Resource & Research Center, Korea Research Institute of Bioscience and Biotechnology, Cheongju 28116, Chungbuk, Republic of Korea; 5 Department of Companion Animal Industry, Wonkwang University, 460 Iksan-daero, Iksan-si 54538, Jeonbuk, Republic of Korea; 6 Center for Animal Resources Development, Wonkwang University, 460 Iksan-daero, Iksan-si 54538, Jeonbuk, Republic of Korea; 7 Institute for Glycoscience, Wonkwang University, 460 Iksan-daero, Iksan-si 54538, Jeonbuk, Republic of Korea

**Keywords:** cutaneous melanoma, ganglioside, GD3, GD2, ST8SIA1, glycosphingolipid, focal adhesion kinase, immunotherapy, quercetin, natural products

## Abstract

Cutaneous melanoma remains lethal once metastatic despite advances in immune checkpoint blockade and BRAF/MEK-directed therapy. Gangliosides are surface-accessible sialylated glycosphingolipids that regulate membrane organization, adhesion, and signaling, representing potential biomarkers and therapeutic targets. This narrative review evaluated the translational readiness of ganglioside-directed strategies in cutaneous melanoma, without assuming that evidence from other GD2- or GD3-positive cancers is directly transferable. We synthesized melanoma-specific evidence on GD3-linked focal adhesion signaling, p130Cas/paxillin activity, PI3K–AKT and MAPK outputs, and distinct phenotypes associated with GD3, GD2, and GM3. We then applied an evidence-gated framework distinguishing melanoma clinical evidence, melanoma preclinical evidence, cross-tumor translational evidence, and hypothesis-generating claims. Direct contemporary clinical evidence in melanoma remains limited. Antibody, cellular, and biosynthetic enzyme strategies require diagnostic-quality antigen profiling, demonstration of surface accessibility and functional dependency, and prospective monitoring for antigen escape and neurotoxicity. Natural compounds, particularly quercetin, are mechanistic probes or formulation-enabled leads: a 2024 study reported reduced GM3/GD3 abundance and FAK/paxillin/AKT signaling in melanoma cell lines, but high in vitro concentrations and the lack of pharmacokinetic or in vivo validation preclude therapeutic conclusions. We proposed a minimum evidence package and go/no-go criteria for clinical translation, specifying experiments needed to advance ganglioside biology into biomarker-defined melanoma interventions.

## 1. Introduction: An Unmet Need Within Modern Melanoma Therapy

Cutaneous melanoma is biologically heterogeneous and challenging to cure following distant metastasis. Immune checkpoint inhibitors and combinations of BRAF and MEK inhibitors have transformed management, but their durable benefit is not universal. Resistance reflects the convergent rewiring of survival signaling, altered differentiation states, stromal interactions, and immune exclusion. Surface glycans offer a complementary therapeutic space because they integrate intrinsic cellular programs with extracellular recognition. Gangliosides are particularly attractive because their carbohydrate epitopes can be directly targeted by antibodies or engineered immune cells, while their biosynthesis can be perturbed enzymatically [1,2,3,4]. Slow-cycling states, BRAF/MEK treatment, melanoma vaccine development and immunotherapy resistance provide the clinical and biological context for complementary targets [5,6,7,8].

GLOBOCAN 2024 estimated 337,969 new cases of melanoma of the skin and 58,398 related deaths worldwide in 2024, with a world age-standardized incidence rate of 3.1 per 100,000 population [9]. These estimates refer to melanoma of the skin rather than all melanoma sites and provide population-level context, not estimates of eligibility for ganglioside-directed treatment.

This narrative review is framed around the Special Issue “Skin Diseases and Their Associated Complications” and focuses on non-acral cutaneous melanoma; acral, mucosal, and uveal melanoma are outside the scope of this review. We review (i) biosynthetic and regulatory foundations; (ii) molecular mechanisms linking gangliosides to melanoma behavior; (iii) therapeutic approaches and barriers; and (iv) the emerging but preclinical role of natural compounds. The search was updated through 9 September 2026 using scholarly databases and reference chaining, with priority given to mechanistic melanoma studies, peer-reviewed reviews, and studies with defined molecular readouts. Because direct clinical evidence for ganglioside-targeted therapy in cutaneous melanoma remains limited, translational claims are deliberately qualified.

Importantly, this review is directly aligned with the Special Issue “Skin Diseases and Their Associated Complications” because it examines cutaneous melanoma as a high-risk skin malignancy in which metastatic progression, treatment resistance, and therapy-related toxicity represent major clinical complications. By focusing on ganglioside-regulated membrane signaling, antigen-directed therapeutic opportunities, and the pharmacological limitations of natural-compound leads, this review connects molecular skin cancer biology to clinically relevant challenges in preventing progression and improving therapeutic precision.

### 1.1. Review Question and Distinct Contribution

The central question is not simply whether gangliosides are biologically relevant in melanoma but what evidence is required before ganglioside-directed interventions can be considered ready for biomarker-defined clinical testing in cutaneous melanoma. The distinct contribution of this review is its evidence-gated translational framework, which integrates target prevalence and accessibility, biological dependency, exposure plausibility, safety, and adaptive monitoring. This framework was designed to prevent direct transfer of therapeutic claims from neuroblastoma or other GD2/GD3-positive cancers to melanoma without disease-specific validation.

### 1.2. Review Scope and Evidence Framework

This article was prepared as a structured narrative review rather than a registered systematic review or meta-analysis. The literature search was last updated on 9 September 2026. We searched Crossref and OpenAlex using predefined concept blocks and conducted backward and forward reference screening of seminal melanoma and ganglioside studies. Search concepts were combined as follows: (“cutaneous melanoma” OR melanoma) AND (ganglioside OR GD3 OR GD2 OR GM3 OR ST8SIA1 OR ST3GAL5); (ganglioside OR GD2 OR GD3) AND (antibody OR CAR-T OR CAR-NK OR immunotherapy); and (melanoma OR ganglioside) AND (quercetin OR flavonoid OR natural product OR nanomedicine). The search concepts and evidence categories are described here. Reproducible search exports and a complete study-level extraction dataset are not available; therefore, this narrative synthesis should not be interpreted as an exhaustive systematic search.

GM2-directed vaccination and its melanoma clinical evidence are outside the focused synthesis of GD3/GD2/GM3-associated biology and natural-compound leads in this review. GM2 was not included as a dedicated search concept; accordingly, no comprehensive assessment of GM2 vaccine efficacy is claimed. This scope restriction is not evidence that GM2 is clinically unimportant.

Peer-reviewed English-language articles with direct relevance to cutaneous melanoma, ganglioside biology, therapeutic targeting, and pharmacologically interpretable natural compounds were prioritized. For each key study, the disease context, experimental model or patient setting, ganglioside-related endpoints, interventions (where applicable), principal findings, and major limitations were extracted. Evidence was classified as follows: (A) direct clinical evidence in cutaneous melanoma; (B) melanoma-specific preclinical evidence; (C) cross-tumor translational evidence from other GD2/GD3-positive malignancies; or (D) hypothesis-generating/general biological context. Reviews were used for orientation and context, but not as substitutes for primary data from melanoma studies.

This review does not claim systematic review status and does not present a numerical Preferred Reporting Items for Systematic Reviews and Meta-Analyses flow diagram because reproducible database exports and duplicate independent screenings were not available. Instead, transparency is provided through explicit search concepts, eligibility logic, study-level evidence extraction, and a prespecified evidence hierarchy. The text and tables consistently distinguish direct cutaneous melanoma findings from cross-tumor extrapolation and hypothesis-generating statements. This approach is intended to prevent spurious precision while making the basis and limitations of the narrative synthesis auditable.

For transparency, the documented search endpoint is 9 September 2026; a lower publication-date boundary and reproducible database-specific search logs were not recorded. The concept blocks above describe the search approach but do not constitute executable, archived queries. The scope includes primary melanoma studies relevant to ganglioside biology or therapeutic targeting, with reviews used for context. Evidence from other cancers is retained only as explicitly labeled cross-tumor context. Reports without a defined intervention or interpretable experimental endpoint are not used to support melanoma efficacy claims. This scope-based selection is not equivalent to duplicate independent eligibility screening, and selection bias cannot be excluded.

## 2. Ganglioside Biology and Melanoma-Relevant Biosynthesis

Gangliosides contain a ceramide anchor and an oligosaccharide head group bearing one or more sialic acid residues. Their lateral partitioning with cholesterol, receptors, integrins, and kinases supports “glycosynaptic” regulation of signaling. Composition is not a passive lineage marker: changing an individual ganglioside or a glycosyltransferase can alter adhesion, receptor residence, phosphorylation, and downstream transcriptional programs [1,2]. Structural, biosynthetic and analytical overviews provide the biochemical context for this pathway; brain-focused literature is used only to contextualize normal-tissue biology and potential safety concerns, not melanoma efficacy [10,11,12].

**Figure 1:**
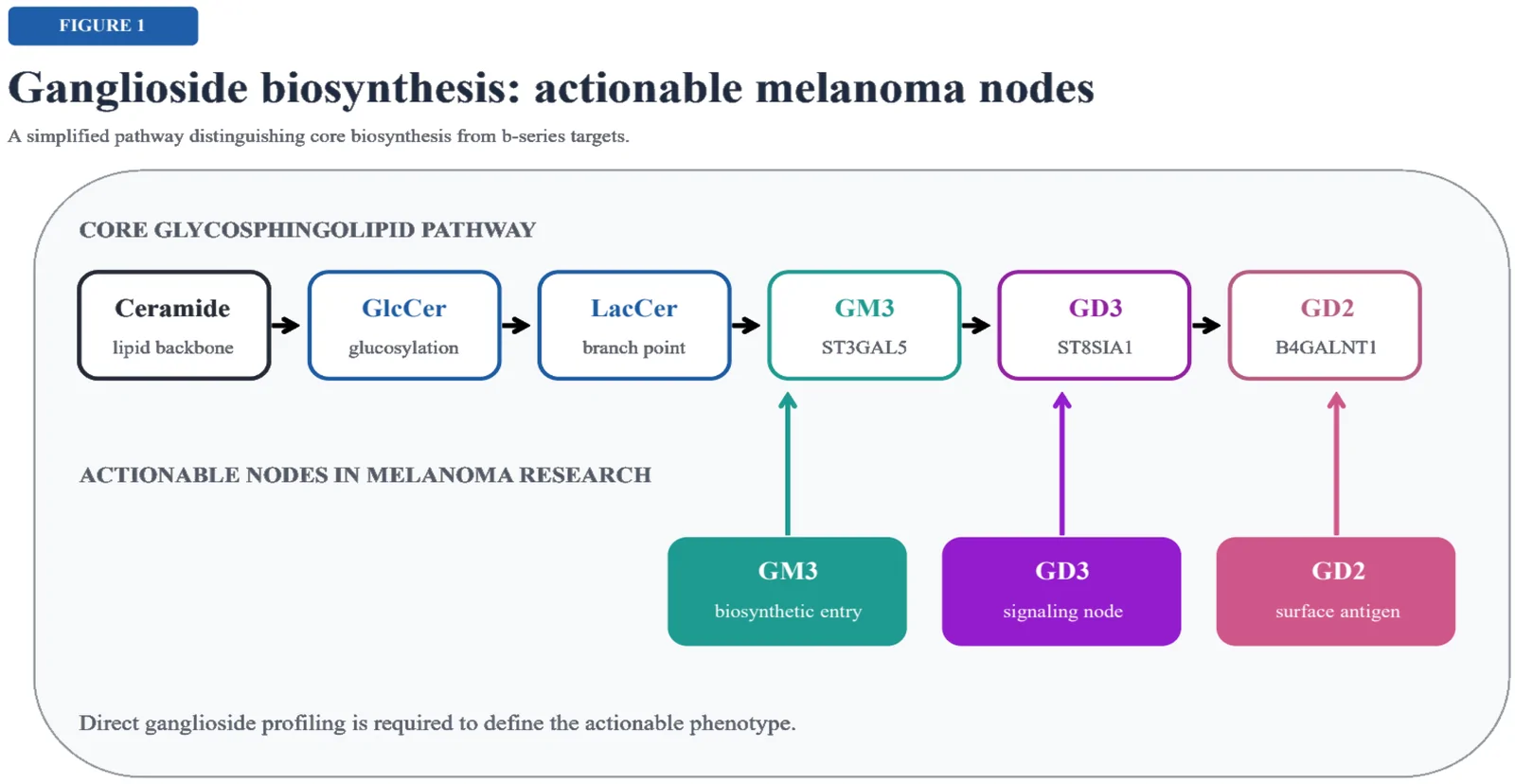
Ganglioside biosynthesis and its principal actionable nodes in cutaneous melanoma. The pathway is conceptual and highlights enzymatic points at which surface antigen composition may be perturbed.

The common pathway begins with ceramides, glucosylceramides, and lactosylceramides. ST3GAL5 (GM3 synthase) produces GM3; ST8SIA1 (GD3 synthase) converts GM3 to GD3, and downstream enzymes generate GD2 and more complex-series structures (Figure 1). These enzymes should be interpreted as flux controllers rather than one-to-one antigen surrogates; substrate availability, competing enzymes, sialidases, and intracellular trafficking all affect the measured cell surface phenotype. This distinction is pivotal when using mRNA or immunoblotting measurements as therapeutic biomarkers. Figure 1 therefore provides the conceptual map for this section: it summarizes the biosynthetic sequence and distinguishes pathway enzymes that control flux from the surface antigens that must be measured directly before therapeutic selection. Table 1 summarizes the biochemical position, melanoma-relevant evidence, and translational implications of the principal gangliosides and biosynthetic nodes.

## 3. Molecular Mechanisms: From Membrane Microdomains to Invasion

The strongest melanoma-specific mechanistic evidence centers on GD3. In GD3-positive derivatives of SK-MEL-28, GD3 enhanced cell growth and invasive properties; p130Cas and paxillin were identified as prominently phosphorylated effectors, and perturbation of these proteins reduced malignant phenotypes [14]. A later comparative model revealed that ganglioside species are not interchangeable: GD3-positive and GD2-positive cells shared some pro-growth properties but differed in adhesion, spreading, migration velocity, and signaling patterns [15]. Thus, statements that “gangliosides promote melanoma” are overly broad; biologic output is species-, context-, and assay-dependent.

**Figure 2:**
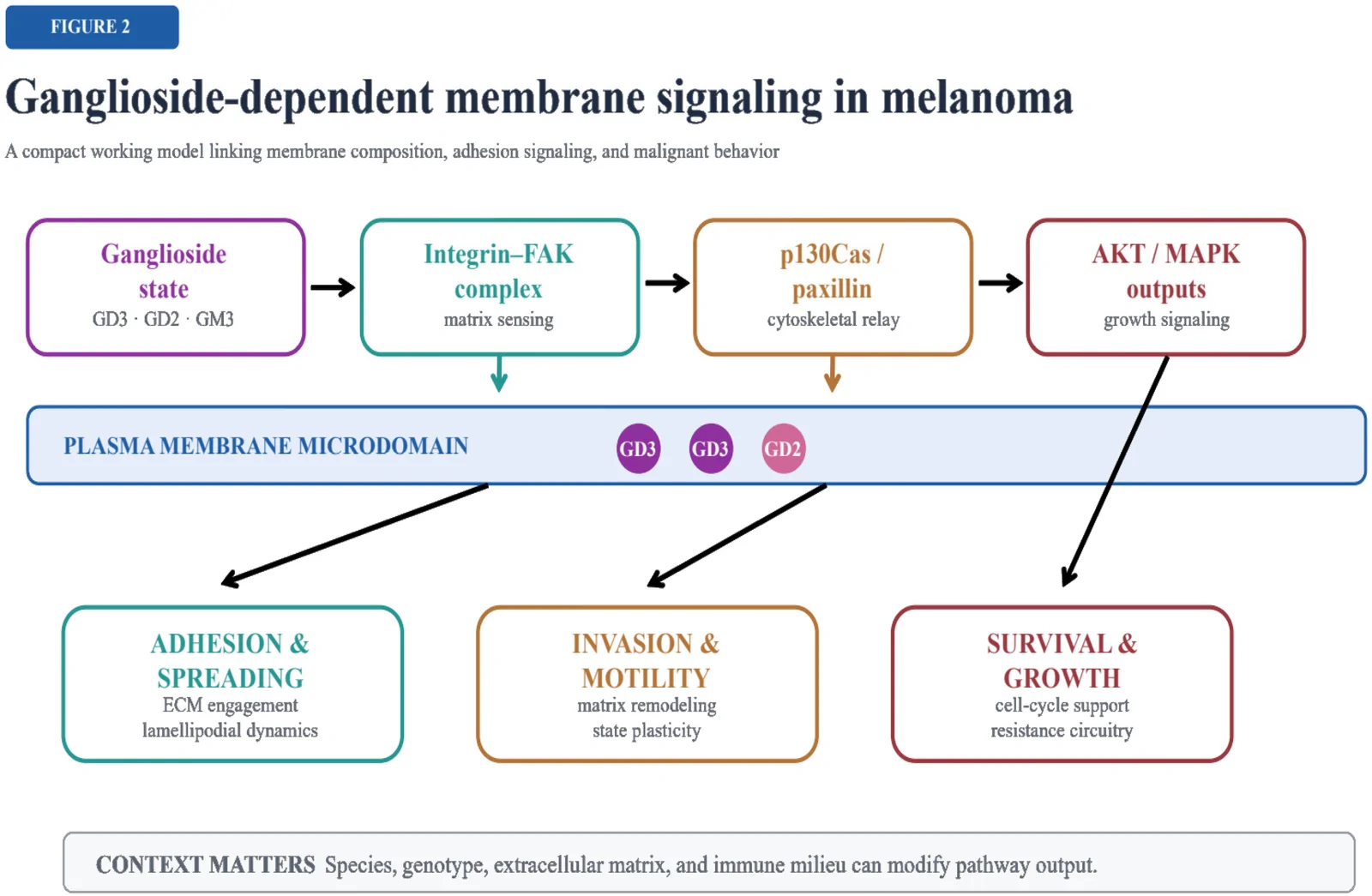
Working model of ganglioside-dependent membrane signaling in cutaneous melanoma. GD3-centered microdomain organization is linked to integrin–FAK–Src, p130Cas/paxillin, and AKT/MAPK outputs; the direction and magnitude of these effects are context-dependent.

A useful working model for gangliosides is to modulate the assembly and signal amplitude of integrin-centered membrane complexes. In melanoma cells, this may engage FAK/Src, p130Cas, and paxillin, which connect extracellular-matrix sensing to cytoskeletal dynamics and PI3K–AKT signaling. These nodes also intersect with RAF–MEK–ERK signaling, cell-cycle progression, survival, and epithelial/mesenchymal-like plasticity. Nevertheless, pathway directionality cannot be inferred from lipid abundance alone. Definitive causality requires gain- and loss-of-function experiments, rescue using defined ganglioside species, and orthogonal lipidomic validation (Figure 2). Figure 2 consolidates this proposed membrane-signaling architecture and should be read as an evidence-weighted working model, not as proof that all GD3-associated effects occur uniformly across melanoma subtypes. FAK and integrin pathway reviews support the broader signaling context, without establishing that every pathway effect in melanoma is initiated by GD3 [17,18].

Gangliosides may also act beyond the tumor cell membrane. Shed gangliosides and ganglioside-bearing extracellular vesicles have the potential to shape myeloid, natural killer (NK), and T-cell functions, although direct melanoma-specific causal datasets remain less mature than the general cancer literature. Their sialylated glycans are plausible contributors to immunoregulatory receptor networks. However, this hypothesis requires experiments that preserve the spatial and compositional complexity of the tumor microenvironment. Distinguishing cell-associated antigens from released glycolipids is particularly important for interpreting antibody accessibility and pharmacodynamic assays. Extracellular-vesicle and tumor-microenvironment reviews provide general context for these hypotheses, rather than direct proof of a melanoma ganglioside-specific immune mechanism [19,20].

### Sialoglycan-Immune Interfaces and the Melanoma Microenvironment

Gangliosides should be considered not only as tumor-cell membrane constituents but also as potential organizers of the tumor–immune interface. Sialylated glycans displayed on lipids and glycoproteins can engage sialic-acid-binding immunoglobulin-like lectins (Siglecs), many of which contain inhibitory signaling motifs in myeloid and lymphoid cells. Across tumor settings, enhanced sialoglycan–Siglec signaling has been associated with an immunosuppressive microenvironment; however, the ligand source, receptor specificity, spatial organization, and relative contribution of glycolipids versus glycoproteins are context-dependent [21]. Thus, a general sialic-acid/Siglec rationale must not be represented as proof that a particular melanoma ganglioside suppresses immunity in patients. In cutaneous melanoma, the relevant question is whether a defined, surface-accessible ganglioside state co-localizes with—and functionally alters—specific immune-cell populations in treatment-naïve and treated lesions. Reviews of tumor-associated macrophages and myeloid-derived suppressor cells describe broader immunosuppressive pathways that could confound an attribution of immune dysfunction specifically to gangliosides [22,23,24]. Immune-cell glycosphingolipid biology, macrophage targeting and neoantigen or mutational-burden frameworks provide context for immune profiling [25,26,27].

The distinction between membrane-bound and shed gangliosides is also operationally important. Released glycolipids and extracellular-vesicle-associated material may affect antigen measurements, antibody bioavailability, and ex vivo immune assays differently from cell-surface antigens. Accordingly, melanoma studies should combine spatial tissue profiling with matched flow-cytometric surface quantification, targeted lipidomics, and functional co-culture assays using autologous or clinically relevant effector cells. Such designs can test whether antigen-high states are associated with altered NK-cell killing, T-cell activation, myeloid-cell polarization, or resistance to checkpoint blockade, rather than inferring these outcomes from bulk transcript abundance alone. The resulting evidence would establish whether gangliosides are actionable immune-modulatory determinants or simply correlate with a broader state of tumor plasticity. Melanoma lipidomics and membrane-metabolism reviews motivate orthogonal profiling, while ion-mobility mass spectrometry provides an analytical precedent for characterizing human melanoma gangliosides [28,29,30,31]. Table 2 summarizes the melanoma-relevant mechanistic evidence, its confidence level, and the experiments required to distinguish association from causality.

## 4. Therapeutic Targeting of Melanoma Gangliosides

### 4.1. Evidence-Gated Framework for Translational Claims

For a ganglioside-directed strategy to advance from a mechanistic rationale to a melanoma clinical study, five evidence gates should be met: (1) analytical validity, requiring reliable, orthogonally confirmed measurement of GD3, GD2, GM3, and relevant biosynthetic enzymes; (2) target prevalence and accessibility, requiring surface density, intratumoral heterogeneity, and spatial distribution in contemporary melanoma specimens; (3) biological dependency, requiring perturbation and rescue experiments demonstrating that the target contributes to the relevant phenotype; (4) exposure and safety plausibility, requiring pharmacologically credible target engagement together with explicit assessment of neural and other on-target/off-tumor risks; and (5) clinical feasibility, requiring biomarker-defined eligibility, on-treatment monitoring, and an antigen-escape plan. Failure at any earlier gate should preclude escalation based solely on favorable evidence from other tumor types.

Direct antigen targeting is conceptually attractive because gangliosides are displayed on the outer membrane leaflet. Anti-GD2 antibodies can drive antibody-dependent cellular cytotoxicity, complement-mediated lysis, and direct cellular effects in GD2-positive tumors [16]. However, the clinical success of GD2-directed treatment in neuroblastoma should not be extrapolated uncritically to melanoma. Melanoma requires rigorous assessment of the target prevalence, uniformity, antigen density, and accessibility in contemporary biopsies, including lesions obtained after immunotherapy or mitogen-activated protein kinase (MAPK)-directed treatment.

Cellular therapies, including chimeric antigen receptor T-cell (CAR-T) and chimeric antigen receptor NK (CAR-NK) platforms, may overcome certain limitations of passive antibody exposure; however, their effectiveness depends on sustained antigen expression and a permissive tumor microenvironment. GD2 is also expressed in peripheral neural tissues, creating a biologically plausible risk factor for neuropathic toxicity. Approaches under consideration for GD2-positive cancers include affinity tuning, transient expression, logic-gated recognition, local delivery, and step-up dosing. For cutaneous melanoma, these approaches should be viewed as design principles rather than established interventions.

Indirect strategies target the biosynthetic machinery. ST8SIA1 is compelling because it regulates flux toward GD3/GD2, and genetic perturbation of this node has been associated with antitumor activity in several cancer systems [4]. Potential advantages include the simultaneous reduction of multiple oncogenic gangliosides, whereas limitations include network compensation, effects on normal tissues, and the current lack of clinically mature selective small-molecule inhibitors. RNA-based suppression, CRISPR-based functional modeling, and targeted protein degradation are research tools or future concepts, not standard**-**of-care approaches.

### 4.2. What Constitutes Direct Clinical Evidence in Cutaneous Melanoma

Direct clinical evidence for ganglioside-directed interventions in cutaneous melanoma remains limited and requires precise characterization. Historical melanoma studies have established the immunogenicity and biological rationale of GD3-directed vaccination and antibody approaches but have not established a contemporary therapeutic standard [32,33]. In contrast, contemporary evidence on GD2 antibodies and GD2-CAR therapy is derived primarily from neuroblastoma and other GD2-positive malignancies [4,16,34,35,36,37,38,39]. Therefore, this review does not claim clinical efficacy of GD2 or GD3 targeting in cutaneous melanoma; rather, it defines a testable translational proposition requiring contemporary biopsy-defined studies.

A minimum clinical validation package should include standardized antigen assays for primary and metastatic lesions; concordance between tissue staining and accessible surface density; associations with genotype, treatment exposure, and immune context; and prospective on-treatment monitoring of antigen modulation. Public melanoma genomic and transcriptomic datasets, including The Cancer Genome Atlas Skin Cutaneous Melanoma (TCGA-SKCM) and Gene Expression Omnibus (GEO), as well as single-cell and spatial datasets, are valuable for hypothesis generation. However, this review did not include *de novo* patient-level bioinformatics analysis. Accordingly, this review does not independently validate unverified claims regarding ST8SIA1/ST3GAL5 survival associations, BRAF/NRAS subgroup effects, immune infiltration correlations, or primary-versus-metastatic differences.

### 4.3. Therapeutic Modality Selection: Lessons That Require Melanoma-Specific Testing

The historical melanoma vaccine literature shows that GD3-directed immunization can elicit measurable antibody responses, but it does not establish clinical benefit in the current era of checkpoint inhibition and molecularly targeted therapy. In a randomized study of 24 melanoma patients, sequential vaccination with GD3-lactone–KLH and the anti-idiotypic antibody BEC2 generated detectable anti-GD3 antibodies in 10 patients, illustrating both biological feasibility and the limited immunogenicity of this target [33]. A contemporary melanoma development program should therefore define whether a ganglioside-directed vaccine is intended to induce binding antibodies, antibody-dependent effector functions, T-cell help, or a combination with checkpoint blockade—and should predefine pharmacodynamic and clinical decision criteria.

GD2-directed CAR-T therapy demonstrates the importance of antigen density, anatomic context, and proactive toxicity management, but the strongest clinical data remain outside melanoma. In an early clinical report in H3K27M-mutated diffuse midline glioma, GD2-CAR T-cell administration produced clinical and radiographic improvement in three of four patients; the toxicities observed were strongly influenced by the vulnerable brainstem disease location [40]. This result should be interpreted as cross-tumor feasibility evidence, not as efficacy evidence for melanoma. In melanoma, candidate products must be tested for trafficking into metastatic sites, persistence, activity in an immunosuppressive microenvironment, and recognition of heterogeneous antigen expression.

Modality choice should be linked to the biology of the intended target. Antibodies require sufficiently dense and accessible surface antigen, appropriate Fc-effector engagement, and a plan for soluble or shed antigen. CAR-T or CAR-NK approaches additionally require durable infiltration, adequate persistence, and resistance to local suppression. These are recognized barriers for solid-tumor cellular therapy generally [41]. For GD2/GD3-directed products, safety packages should include normal-tissue expression mapping, sensory and autonomic neurologic monitoring, dose-escalation rules, and evaluation of affinity-tuned, transient, local, or logic-gated designs where justified. A negative or heterogeneous antigen profile should redirect development toward biosynthetic or downstream pathway perturbation rather than escalating an antigen-directed product. Table 3 compares the evidence, putative mechanisms, potential advantages, and key limitations of antibody-, cell-, and biosynthesis-directed strategies.

## 5. Natural Compounds: Opportunity, Evidence Boundaries, and Quercetin

Natural products have historically contributed useful anticancer scaffolds; however, natural origin does not establish target specificity, systemic exposure, or clinical efficacy. In melanoma, polyphenols, such as quercetin, resveratrol, and curcumin, have been reported to influence redox homeostasis, mitochondrial apoptosis, inflammatory signaling, and proliferation in preclinical studies. Nevertheless, their translation is constrained by low aqueous solubility, variable metabolism, poor oral bioavailability, and the frequent use of supraphysiological concentrations in cell culture [42,43,44]. Consequently, natural compounds can be framed as mechanistic probes, lead structures, or formulation-enabled partners. Earlier melanoma experiments with quercetin and genistein provide cell-based context, not proof of a ganglioside-dependent mechanism [45,46,47]. Broader phytochemical reviews likewise cannot establish melanoma efficacy [48].

A 2024 study published in IJMS provides a focused starting point [13]. In SK-MEL-28 and G-361 cells, quercetin reduced viability, increased G0/G1 arrest and mitochondrial apoptosis markers, impaired wound closure, and decreased GM3/GD3 abundance and ST3GAL5/ST8SIA1 expression. The authors attributed these changes to reduced FAK/paxillin/AKT signaling. This is an important hypothesis-generating observation because it connects a bioactive flavonoid to a tractable glycolipid pathway. However, the measured half-maximal inhibitory concentrations were approximately 268–272 µM, and the mechanistic experiments commonly used 100–300 µM. These concentrations substantially limit direct clinical extrapolation. Because cytotoxicity can secondarily suppress metabolic and biosynthetic programs, temporal ordering and rescue experiments are needed to establish whether GD3 reduction is a primary mediator of quercetin action.

Subsequent experiments should be rigorous and include: (i) time-resolved targeted lipidomics before overt loss of viability; (ii) rescue with exogenous GD3 or enforced ST8SIA1 expression; (iii) comparison with FAK and AKT inhibitors; (iv) testing in BRAF-mutant, NRAS-mutant, and triple-wild-type patient-derived cultures; (v) evaluation in 3D, immune-competent, or humanized systems; and (vi) pharmacokinetic/pharmacodynamic studies of a formulation achieving clinically plausible exposure. Such investigations would clarify whether quercetin directly modulates the ganglioside pathway or acts as an upstream stressor that converges on it. Table 4 summarizes the natural-compound evidence and distinguishes reported anti-melanoma phenotypes from validated ganglioside-dependent mechanisms.

### 5.1. Pharmacological Boundaries for Natural-Compound Translation

Based on the accumulated evidence, quercetin is best considered a ganglioside-modulating chemical probe and a possible formulation-enabled combination lead, rather than a stand-alone therapeutic candidate for patients with melanoma. In the source study, the reported IC50 values were 271.87 μM and 268.39 μM in SK-MEL-28 and G-361 cells, respectively [13]. Such high micromolar concentrations require careful interpretation because exposure achieved in culture may not be safely or sustainably attainable in human tumors. Any conclusion that GD3 suppression is a primary mechanism requires testing before substantial loss of cell viability, using quantitative lipidomics and rescue experiments.

Systemic translation is also constrained by poor aqueous solubility, limited oral bioavailability, extensive phase II conjugation (including glucuronidation), rapid metabolism, and uncertain tumor penetration [42,43,50,51,52]. Nanocarriers, prodrugs, local delivery, or coformulations may improve exposure; however, they introduce additional requirements for biodistribution, stability, toxicology, and pharmacodynamic confirmation. Accordingly, development should proceed by first demonstrating target engagement at pharmacologically plausible concentrations, then establishing causality through ST8SIA1/ST3GAL5 perturbation and ganglioside rescue, evaluating rational combinations, and finally assessing in vivo efficacy.

Intervention identity must be preserved when interpreting natural-compound evidence. Unmodified quercetin (aglycone), quercetin glycosides, chemically modified derivatives or prodrugs, combination regimens, and carrier-based formulations are distinct interventions; an effect reported for one should not be attributed automatically to free quercetin. Formulation or combination studies require identification of the payload, carrier and co-treatment, with appropriate free-compound, empty-carrier and single-agent controls. Exposure comparisons should distinguish parent compound from metabolites and conjugates. The melanoma-cell findings summarized above support only the tested quercetin intervention and do not establish efficacy of a glycoside, derivative or formulation.

### 5.2. Designing Pharmacologically Credible Natural-Compound Studies

Natural-product research is particularly vulnerable to a gap between an in vitro phenotype and a clinically credible intervention. A compound should not be advanced merely because it decreases viability and a ganglioside-associated readout in the same experiment. The concentration–response relationship should be reported for both target engagement and nonspecific cytotoxicity, and samples for mechanistic analyses should be collected at time points preceding substantial apoptosis or loss of cell number. For quercetin, this distinction is essential because the reported anti-melanoma effects and reductions in GM3/GD3-associated biosynthetic markers occurred at high micromolar concentrations [13]. A more convincing study would show that modulation of a defined ganglioside species occurs at an exposure that is separable from general cell stress, and that the phenotype is altered by genetic perturbation or biochemical rescue of the relevant pathway.

Formulation work should be treated as a hypothesis to test, not as a solution presumed to overcome exposure limitations. Nanocarriers, prodrugs, local delivery systems, or rational co-formulations may modify solubility, circulation time, tissue distribution, and intracellular delivery, but each platform can introduce new variables, including carrier toxicity, altered biodistribution, batch variability, and immune interactions. Consequently, an in vivo study should measure parent compound and relevant metabolites in plasma and tumor, establish the temporal relation between exposure and ganglioside modulation, and use a pharmacodynamic assay that is technically independent of the discovery assay. The literature on quercetin repeatedly identifies limited aqueous solubility, metabolism, and low systemic bioavailability as major translational constraints [42,43,50,51,52]. These properties make unsupported dose equivalence between cell culture and patients inappropriate.

Model selection must also reflect melanoma heterogeneity. Candidate compounds should be tested in BRAF-mutant, NRAS-mutant, and triple-wild-type cultures; in models with high and low baseline GD3/GD2/GM3 states; and, where feasible, in three-dimensional and immune-competent systems. Combination studies with BRAF/MEK, FAK, PI3K/AKT, or checkpoint-directed approaches should use formal interaction analyses and be designed to distinguish additive toxicity from restoration of a ganglioside-dependent vulnerability. A negative rescue experiment or a failure to achieve tumor exposure is informative: it would reposition the compound as a mechanistic probe rather than justify escalation to an efficacy claim. This evidence-first approach is compatible with natural-product discovery while maintaining the pharmacological rigor expected for translational melanoma research.

Experimental context and treatment timing are separate evidence dimensions. Two-dimensional cell cultures, patient-derived cultures, three-dimensional systems and in vivo melanoma models should not be pooled as interchangeable efficacy evidence. Compound exposure before a challenge or tumor establishment tests prevention or sensitization, whereas treatment of established disease addresses a therapeutic question; neither cell-culture viability nor pretreatment alone demonstrates regression of an established melanoma. Future melanoma studies should report model identity, genotype, immune competence, disease stage at treatment initiation, dosing schedule and exposure.

## 6. Biomarker-Led Translational Roadmap

**Figure 3:**
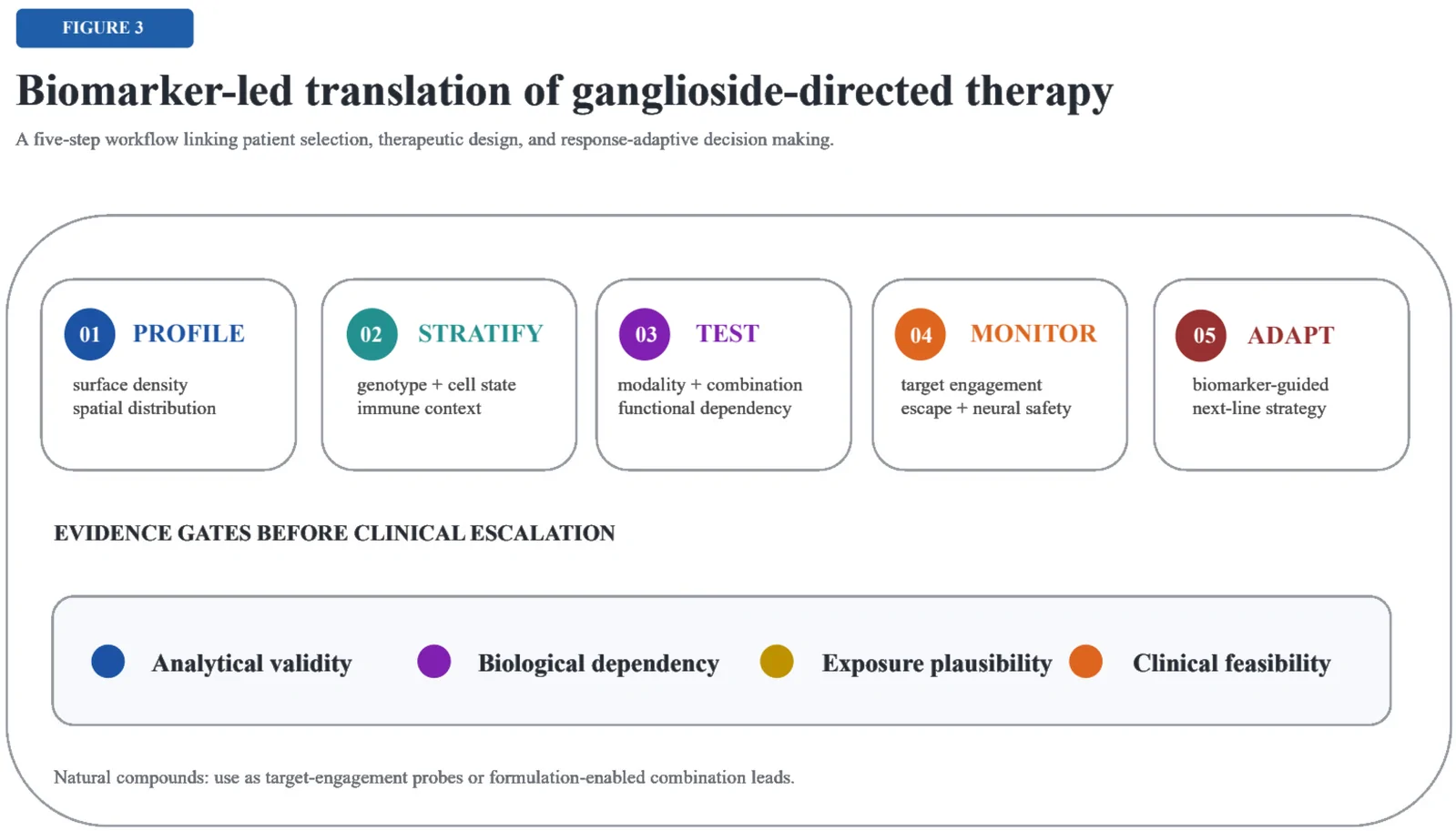
Biomarker-led translational workflow for ganglioside-directed strategies in cutaneous melanoma. The pipeline emphasizes patient-level profiling, biological dependency, clinically plausible exposure, and on-treatment monitoring.

The field should move beyond single-marker enthusiasm toward an integrated, evidence-gated decision framework (Figure 3). First, patient specimens should undergo standardized, orthogonal assessment of GD3, GD2, GM3, and relevant biosynthetic enzymes, combining immunohistochemistry or imaging mass spectrometry for spatial localization, flow cytometry for accessible surface density, and targeted glyco/lipidomics for chemical identity. Second, ganglioside profiles should be evaluated in relation to driver genotype, differentiation state, immune infiltration, prior therapy, and clinical outcomes. Third, functional experiments should determine whether antigen-high and antigen-low tumors differ in signaling dependencies, therapeutic vulnerabilities, and potential escape mechanisms. Figure 3 integrates these requirements into a sequential decision pathway: it shows that analytical validity, biological dependency, exposure plausibility, and clinical feasibility are linked gates rather than interchangeable indicators of translational readiness.

Clinical development should begin with a diagnostic-quality assay and prespecified antigen thresholds rather than an assumption of uniform positivity. Early studies need on-treatment biopsies and blood-based monitoring to quantify antigen modulation, soluble or shed gangliosides, immune activation, and neural toxicity. Combination trials should be supported by a falsifiable mechanistic rationale, such as FAK-pathway dependency or complement/effector-cell recruitment, and should incorporate prospective monitoring for antigen-negative relapse. Natural compounds should be considered only when formulation, systemic exposure, and a validated target-engagement assay are prospectively defined.

### 6.1. From Exploratory Signal to a Clinical-Grade Biomarker Strategy

A clinically useful ganglioside biomarker cannot be inferred from a single assay or from biosynthetic-enzyme expression alone. A stepwise strategy should begin with analytical validation: pre-analytical tissue handling, antibody specificity, assay linearity, inter-observer reproducibility, and concordance with an orthogonal chemical measurement should be specified before association analyses. Tissue immunohistochemistry or multiplex imaging can define spatial localization; flow cytometry of fresh tissue or patient-derived cultures can estimate accessible surface density; and targeted mass-spectrometric methods can verify molecular species. Spatial lipidomics is particularly relevant because it preserves histopathologic context. In a small patient series, ToF-SIMS imaging identified lipid-profile differences between primary and metastatic melanoma, including increased GM3 gangliosides in metastatic samples; the exploratory sample size underscores the need for independent validation [53].

The second stage is clinical validation in representative cohorts. Primary tumors, locoregional disease, and distant metastases should be sampled where feasible, with annotation of BRAF/NRAS/NF1 status, differentiation state, prior systemic therapy, and immune contexture. Rather than dichotomizing a marker at an arbitrary threshold, investigators should assess the distribution of antigen density, intralesional heterogeneity, interlesional discordance, and longitudinal change under treatment. The endpoint should be prespecified—for example, target-mediated internalization, antibody occupancy, change in a ganglioside species, or association with a defined resistance phenotype—so that exploratory correlations are not mislabeled as predictive biomarkers.

The third stage is a decision-ready trial design. Early studies should enroll only patients whose lesions meet a prospectively defined, assay-qualified antigen criterion and should include baseline and on-treatment sampling whenever clinically appropriate. A minimum data package includes target density and accessibility, evidence of biological dependency in matched models, pharmacokinetic exposure relative to an active concentration, pharmacodynamic target engagement, and active surveillance for antigen-negative escape and neurologic adverse events. This framework does not delay translation unnecessarily; it reduces the risk of interpreting an underexposed, antigen-low, or biologically nondependent tumor as a failure of the therapeutic class.

### 6.2. Priorities for an Efficient Translational Evidence Package

For an efficient first translational package, investigators should avoid collecting disconnected descriptive datasets. A focused program could profile matched primary and metastatic specimens, use a prespecified panel of GM3, GD3, GD2, ST3GAL5, and ST8SIA1 measurements, and then select a limited set of antigen-high and antigen-low models for perturbation experiments. Evidence of causal dependency should be sought through genetic and pharmacologic loss-of-function approaches, followed by rescue with defined ganglioside species or restoration of the relevant biosynthetic enzyme. If the phenotype is not rescued, the ganglioside change may be a correlated consequence of cellular stress rather than the driver of therapeutic sensitivity.

The same discipline should guide combination development. A ganglioside-directed agent should be combined with a checkpoint inhibitor, MAPK-pathway inhibitor, FAK/PI3K inhibitor, or effector-cell strategy only when the combination has a testable mechanistic rationale and a biomarker-based enrichment hypothesis. Study protocols should distinguish pharmacodynamic success (for example, target reduction or receptor occupancy), biological success (reduced growth, invasion, or immune suppression), and preliminary clinical activity. Clear separation of these levels of evidence will make negative results interpretable and will help ensure that a promising but incompletely validated glycolipid signal is not advanced as a therapeutic conclusion.

## 7. Conclusions

Gangliosides represent potential biological and therapeutic determinants in melanoma, but their clinical translation warrants an evidence-gated approach. Melanoma-specific evidence supports a functional role for GD3-centered membrane signaling in growth, invasion, and adhesion-related pathways, whereas GD2 and GM3 appear to be associated with partially distinct cellular programs. The principal contribution of this review is to distinguish mechanistic plausibility from translational readiness: antigen presence or cross-tumor precedent alone is insufficient to justify clinical development. Candidate modalities must satisfy analytical, biological, pharmacological, safety, and clinical-feasibility criteria in biomarker-defined cutaneous melanoma.

Future research should prioritize standardized antigen profiling across diverse melanoma subtypes, including BRAF-mutant, NRAS-mutant, and triple wild-type tumors. Integrating ganglioside measurements with immune infiltration, treatment history, and clinical outcomes may clarify whether these glycolipids function as predictive biomarkers or identify therapeutic vulnerabilities. Clinical translation will also require explicit strategies to mitigate neural toxicity, given the GD2 expression in peripheral tissues. Rational drug combinations, such as ganglioside suppression with FAK/PI3K/MAPK inhibitors, should be tested in biomarker-defined cohorts using prospectively defined mechanistic hypotheses rather than an empirical trial design. Natural compounds such as quercetin may serve as chemical probes to dissect ganglioside-dependent signaling; however, appropriate formulation, pharmacokinetic, and target-engagement validation strategies are needed before clinical application.

In conclusion, gangliosides represent a biologically relevant but complex therapeutic axis in cutaneous melanoma. Their translation into clinically actionable strategies will depend on rigorous biomarker development, mechanistic validation, and innovative therapeutic design that balances efficacy and safety. By integrating molecular profiling, functional perturbation, and early-phase clinical studies, this field can determine whether ganglioside biology can support precise and durable therapeutic strategies in melanoma.

## Figures and Tables

**Table 1:** This table complements Figure 1 by translating this pathway map into a target-by-target evidence summary. It enables the reader to compare biochemical position, melanoma-specific support, and the distinct biomarker or intervention question associated with each ganglioside-related node.

Ganglioside/Node	Biochemical Position	Melanoma-Relevant Evidence	Translational Implication
GM3/ST3GAL5	Initial sialylation of lactosylceramide	Detected with GD3 in melanoma models; altered by quercetin in two cell lines [13].	Profile jointly with downstream species; isolated GM3 effects may differ by model.
GD3/ST8SIA1	Disialylated product of GM3	GD3 increases growth/invasion in engineered melanoma systems and activates p130Cas/paxillin-associated programs [14,15].	Candidate antigen and biosynthetic target; validate abundance spatially.
GD2	Downstream b-series ganglioside	Supports strong adhesion in a melanoma model; widely studied target in other GD2-positive tumors [15,16].	Potential antibody/cell-therapy antigen, limited by neural-tissue expression.
Ceramide balance	Shared lipid backbone	Ceramide metabolites can modify apoptosis and stress signaling.	Combination rationale; requires lipidomic rather than transcript-only assessment.

**Table 2:** This table separates the mechanistic observations summarized in Figure 2 by confidence level and identifies the experiment needed to move each association from mechanistic plausibility toward causal inference. Together, the figure and table emphasize where melanoma-specific evidence is strong and where it remains provisional.

Mechanistic Layer	Representative Observations	Confidence	Key Experimental Need
Membrane organization	Gangliosides partition into receptor-rich lipid microdomains [1,2].	High (general biology)	Super-resolution imaging/spatial lipidomics in patient melanoma.
Adhesion signaling	GD3-associated p130Cas/paxillin phosphorylation promotes growth/invasion in melanoma models [14].	High (model-specific)	Validation across genotype- and state-diverse patient-derived models.
Phenotypic specificity	GD3, GD2, GM3, GM2, and GM1 create nonidentical melanoma phenotypes [15].	High (model-specific)	Quantitative surface antigen and glycomics correlation.
Immune modulation	Tumor-associated gangliosides can influence immune recognition in cancer [3,4].	Moderate for melanoma	Autologous co-culture and spatially resolved human-tumor studies.

**Table 3:** This table provides a modality-level synthesis of this section. Rather than ranking approaches by theoretical appeal alone, it compares the evidence level, putative mechanism, anticipated advantage, and decisive limitation for antibody-, cell-, and biosynthesis-directed strategies, thereby clarifying the experiment or safety package required before melanoma-specific escalation.

Approach	Evidence Level	Putative Mechanism	Potential Advantage	Key Limitation/Next Step
Anti-GD2 antibodies	Clinical experience in other GD2-positive tumors; melanoma-specific clinical benefit remains unestablished.	GD2 surface binding; Fc/complement-mediated killing.	Clinically developed extracellular target in other cancers.	Establish melanoma antigen prevalence/density and efficacy; assess peripheral-neural toxicity.
Anti-GD3 antibodies	Melanoma-specific clinical experience with R24 [32]; efficacy evidence remains limited and is not equivalent to established clinical benefit.	GD3 surface binding; Fc/complement-mediated killing.	Direct historical clinical investigation in melanoma.	Validate efficacy, antigen accessibility and safety in contemporary biomarker-defined melanoma studies.
CAR-T/CAR-NK	Other solid tumors; hypothesis-generating in melanoma.	Engineered antigen recognition and cytotoxicity.	Modular and potentially high potency.	Evaluate trafficking, persistence, antigen escape, and on-target/off-tumor effects in melanoma models.
ST3GAL5/ST8SIA1 suppression	Melanoma-specific preclinical for pathway relevance; broader cancer support.	Reduces flux toward GM3/GD3/GD2.	Could modulate several gangliosides simultaneously.	Development of selective, clinically usable agents and evaluation of normal-tissue safety.
FAK/PI3K/MAPK combinations	Melanoma-specific mechanistic rationale; clinical hypothesis.	Targets downstream adhesion/survival nodes.	May counter incomplete antigen dependency.	Use prespecified ganglioside and pathway biomarkers in combination-treatment studies.

**Table 4:** This table is intended as a translational filter for the natural-compound literature. It distinguishes a reported anti-melanoma phenotype from a demonstrated ganglioside mechanism and highlights the exposure, formulation, and in vivo evidence required before a compound can be viewed as more than a mechanistic probe.

Compound/Class	Reported Preclinical Actions	Ganglioside Link	Translation Status and Caveat
Quercetin	Cell-cycle arrest, mitochondrial apoptosis, reduced migration in melanoma models [13].	Reduced GM3/GD3 and ST3GAL5/ST8SIA1 expression in two cell lines [13].	Preclinical; high micromolar exposures and in vivo validation gap.
7,8-Dihydroxyflavone	Mitochondrial apoptosis and anti-melanoma activity.	Downregulated GD3 expression in melanoma cells [49].	Preclinical; requires independent replication and exposure data.
Resveratrol/curcumin	Pleiotropic redox, inflammatory, and survival-pathway effects [43].	No established melanoma-specific ganglioside mechanism.	Ganglioside targeting should not be inferred from broad anticancer activity.
Formulation-enabled polyphenols	Nanocarriers/prodrugs may improve delivery.	Potential to assess ganglioside-linked pharmacodynamics.	Carrier safety, biodistribution, and clinical benefit remain to be established.

## Data Availability

No new data were created or analyzed in this study. Data sharing is not applicable to this article.
